# Disease Models & Mechanisms: a short history of supporting early-career researchers

**DOI:** 10.1242/dmm.052796

**Published:** 2025-12-29

**Authors:** Rachel Hackett

**Affiliations:** The Company of Biologists, Bidder Building, Station Road, Histon, Cambridge CB24 9LF, UK

## Abstract

The scope of Disease Models & Mechanisms (DMM) encompasses different communities – clinicians, basic researchers, translational scientists, patients and their advocates and families, as well as many different disease areas and model systems used to interrogate mechanism and possible therapy. Since its launch, DMM has maintained a commitment to supporting the next generation of scientists, supported by its publisher, the not-for-profit The Company of Biologists. Such initiatives include First Person interviews, Conference Travel Grants, Travelling Fellowships and the annual Outstanding Paper Prize. In this Editorial, we trace the history of the support offered by DMM, with a particular focus on the hugely popular Conference Travel Grants, and highlight testimonials from grant recipients.

## Teaser

**Summary:** This Editorial outlines the ways in which DMM has supported early-career researchers over the years, with a particular focus on Conference Travel Grants.

## Travelling Fellowships

Between 2008 and 2025, four of The Company of Biologists’ journals – Development, Journal of Cell Science (JCS), Journal of Experimental Biology (JEB) and Disease Models & Mechanisms (DMM) – offered Travelling Fellowships of up to £3000 to enable early-career researchers (ECRs) to make collaborative visits to other laboratories. These fellowships have enabled researchers from all over the world within the community of the Company journals to gain cutting-edge research experience, expand their professional networks and publish their research – helping to develop their careers, with some former recipients establishing their own labs later in life (Adhikari, 2025a,b,c).

During the past decade (2015–2025), Development, JCS, JEB and DMM have collectively awarded Travelling Fellowships to 841 ECRs, selected from a total of 2244 applicants. Each year, the Company typically receives around 300–400 applications, which are then assessed by a research-active academic editor based on the quality of the proposal and the potential impact of the visit. The selection process also ensures that fellowship recipients represent a diverse range of genders, ethnicities and geographical locations**.** Since 2015, DMM alone has awarded Travelling Fellowships to 188 ECRs.

## Conference Travel Grants

DMM launched its Conference Travel Grants in 2015. These offer financial support to ECRs wanting to attend in-person and virtual meetings, conferences, workshops and training courses that relate to the scope of the journal. We also welcome applications from independent group leaders and PIs with no independent funding. There are four funding rounds a year to accommodate the busy conference calendars and give applicants flexibility. One of the challenges for DMM is that our journal has no core society, (fully) relevant annual conference or single community that it can target for support, so our Conference Travel Grants are vital in helping to further support those working within our broad range of communities.

Over the past 10 years, we have awarded 885 Conference Travel Grants to applicants from around the world. To build a truly global scientific community, these grants have no geographic, citizenship or residency restrictions for applicants, and there are no limits on the destination country. DMM has aided ECRs to attend conferences covering a breadth of subject areas, as can be seen in Box 1, which names some of the conferences that Conference Travel Grant recipients have attended during 2025 alone, representing travel to 35 countries from Argentina to Uruguay for 189 successful applicants.

Box 1. (Non-exhaustive) list of conferences, meetings and workshops attended by recipients of DMM Conference Travel Grants in 2025

- International Society for Experimental Hematology 54th Annual Scientific Meeting
- 2nd International Society for Extracellular Vesicles (ISEV) International Conference on Extracellular Vesicles in Nervous Systems
- 10 years of mesoSPIM Symposium
- ‘Organoids: modelling organ development and disease in 3D’ – European Molecular Biology Organization (EMBO) | European Molecular Biology Laboratory (EMBL) Symposium
- European Organisation for Research and Treatment of Cancer (EORTC) Cutaneous Lymphoma Tumour Group Annual Meeting 2025
- Society for Melanoma Research (SMR) 22nd International Congress
- ‘Angiogenesis and Social Interactions with Neighboring Cells and Tissues in Health and Disease’ Gordon Research Conference
- 6th edition of Metabolism and Cancer Conference
- American Society for Human Genetics 2025 Annual Meeting
- International Society for Neurochemistry (ISN) 2025 Meeting
- Federation of Clinical Immunology Societies (FOCIS) European Advanced Course and Conference on Immunology and Immunopathology 2025
- International Conference on Microplastic Pollution in the Mediterranean Sea 2025
- International Cardiovascular Development Anatomy and Regeneration Meeting (ICDAR 25)
- 8th edition of the Advanced Epilepsy Course Bridging Basic with Clinical Epileptology 2025
- Zebrafish Disease Models Society Annual Meeting 2025
- 13th World Congress on Alternatives and Animal Use in the Life Sciences 2025
- Targeting RAS Symposium – 2nd edition
- Spinal Research Network Meeting 2025
- 52nd European Muscle Conference
- 25th International Worm Meeting (Genetics Society of America)
- Immuno-Cardiology Symposium (The Jackson Laboratory)
- American Society for Bone and Mineral Research (ASBMR) Annual Meeting
- 9th International Congress on Neuropathic Pain – NeuPSIG 2025
- ‘*Drosophila* genetics and genomics’ EMBO course
- 49th Annual International Herpesvirus Workshop
- Epigenetic Inheritance: Impact for Biology and Society 2025
- International Society for Stem Cell Research (ISSCR) 2025 Annual Meeting
- 19th International Congress of Immunology (IUIS 2025)
- Cortical Development Conference 2025
- NanoMed Europe 2025
- 11th International γδ T cell Conference
- ‘2025 Cancer Genetics and Epigenetics’ Gordon Research Conference and Seminar
- XVII European Meeting on Glial Cells in Health and Disease
- 10th Congress of the BioIron Society
- 22nd International Society for Human and Animal Mycology (ISHAM) Congress 2025
- Computational Immunology Summer School
- ‘Salmonella Biology and Pathogenesis’ Gordon Research Conference
- ‘Chronobiology’ Gordon Research Conference and Seminar
- ‘Central Nervous System Injury and Repair’ Gordon Research Conference
- ‘Germinal Stem Cell Biology: *In Vivo* and *In Vitro* Approaches Toward Fundamental Mechanisms and Diverse Applications’ Gordon Research Conference
- American Association for Cancer Research (AACR) Annual Meeting
- XIXth Negative-strand RNA Virus Meeting
- European Hematology Association (EHA) 2025 Congress
- Digestive Disease Week 2025
- ‘Transposable Elements in the Era of Data Science’ EMBO course
- European Association for Cancer Research (EACR) 2025 Congress: Innovative Cancer Science
- British Association for Cancer Research 65th Anniversary Meeting
- European Network to Cure ALS (ENCALS) 2025
- ‘Cancer Genetics and Epigenetics’ Gordon Research Conference
- European Society for Neurochemistry and Hellenic Society for Neuroscience Meeting 2025 (ESN-HSN 2025)
- Federation of American Societies for Experimental Biology (FASEB) Cellular Plasticity in Cancer
- ‘Translational Machinery in Health and Diseases’ Gordon Research Conference
- ‘Molecular Pharmacology’ Gordon Research Conference
- 17th International Conference of the Society of Neuroscientists of Africa (SONA 2025)
- 3D Cell Culture 2025: Functional Precision Medicine Conference
- 10th Swiss Virology Meeting
- ‘Directed Cell Migration 2025: How Cells Migrate and Sense Direction Under Diverse Physiological Conditions’ Gordon Research Conference
- AD/PD 2025 Alzheimer's & Parkinson's Diseases Conference
- 23rd Groupe des Études des Membranes (GEM) Meeting – Diving into the secrets of cellular membranes
- ‘2025 Cartilage Biology and Pathology’ Gordon Research Conference
- ‘2025 Glycobiology’ Gordon Research Conference and Seminar
- Lorne Infection and Immunity 2025
- 12th African Congress of Immunology

**Fig. 1.**
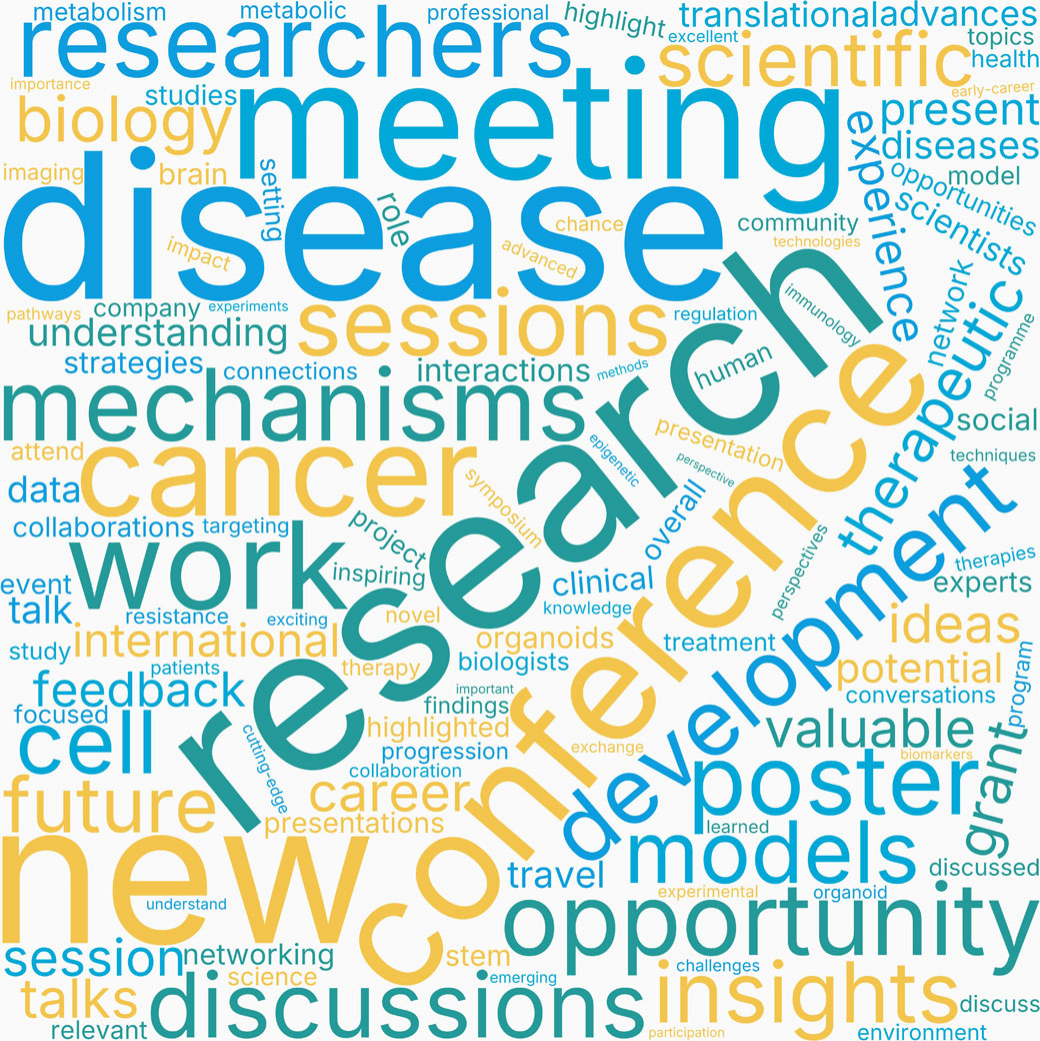
**Word cloud showing a selection of the most frequently used words in reports delivered by recipients of DMM Conference Travel Grants.** Generated with https://www.wordclouds.com/.

The reports our grantees submit after they have returned from their events give us important insight into what junior scientists value most about attending such events (see Fig. 1 for a word cloud based on reports that we have received). For most, it was the opportunity to present their work, in a talk and/or poster.

Xiaozheng Liu, from KU Leuven, Belgium, attended the 6th edition of Metabolism and Cancer Conference and commented “I was honoured to present my research talk titled ‘Targeting the lipid metabolism of alveolar type II cells decreases lung metastasis’ … Presenting at this conference allowed me to receive feedback from world-leading experts in cancer metabolism, refine my research direction and build visibility in the field. I was truly grateful to receive the Best Oral Communication Award, which has significantly strengthened my motivation as an early-career scientist”.

Caroline Carneiro, from the University of New Brunswick, Canada, attended the European Society for Evolutionary Biology conference: “One of the most memorable moments was presenting my poster to one of the leading experts in cancer heterogeneity, who was cited in my work. Our conversation was very helpful and gave me new ideas and guidance for my research. I also attended his presentation on his latest discoveries, which provided more insights and inspired new directions for my project”.

Patrizia Tornabene (Cincinnati Children's Hospital Medical Center, USA) attended the European Molecular Biology Organization (EMBO) | European Molecular Biology Laboratory (EMBL) Symposium ‘Organoids: modelling organ development and disease in 3D’: “In addition to groundbreaking science, the symposium provided several networking opportunities with peers and senior leaders at the forefront of organoid development and application. Finally, thanks to the DMM travel award, I'm glad I was able to present my work at the symposium, where I received insightful feedback and laid the foundation for potential future collaborations”.

For some, conference attendance can expand one's way of thinking. Dr Abolarin Patrick Oluwole, Babcock University, Nigeria, wrote “I am exceedingly grateful to The Company of Biologists for providing me with the DMM Conference Travel Grant that aided my participation in the SONA (17th International Conference of The Society of Neuroscientists of Africa) 2025, Marrakech, Morocco. I am enriched, my experience is broadened, and my perspective on the multifaceted disease models and mechanisms, diagnostic biomarkers and therapeutic strategies in neuropsychiatric research is improved”.

**Fig. 2.**
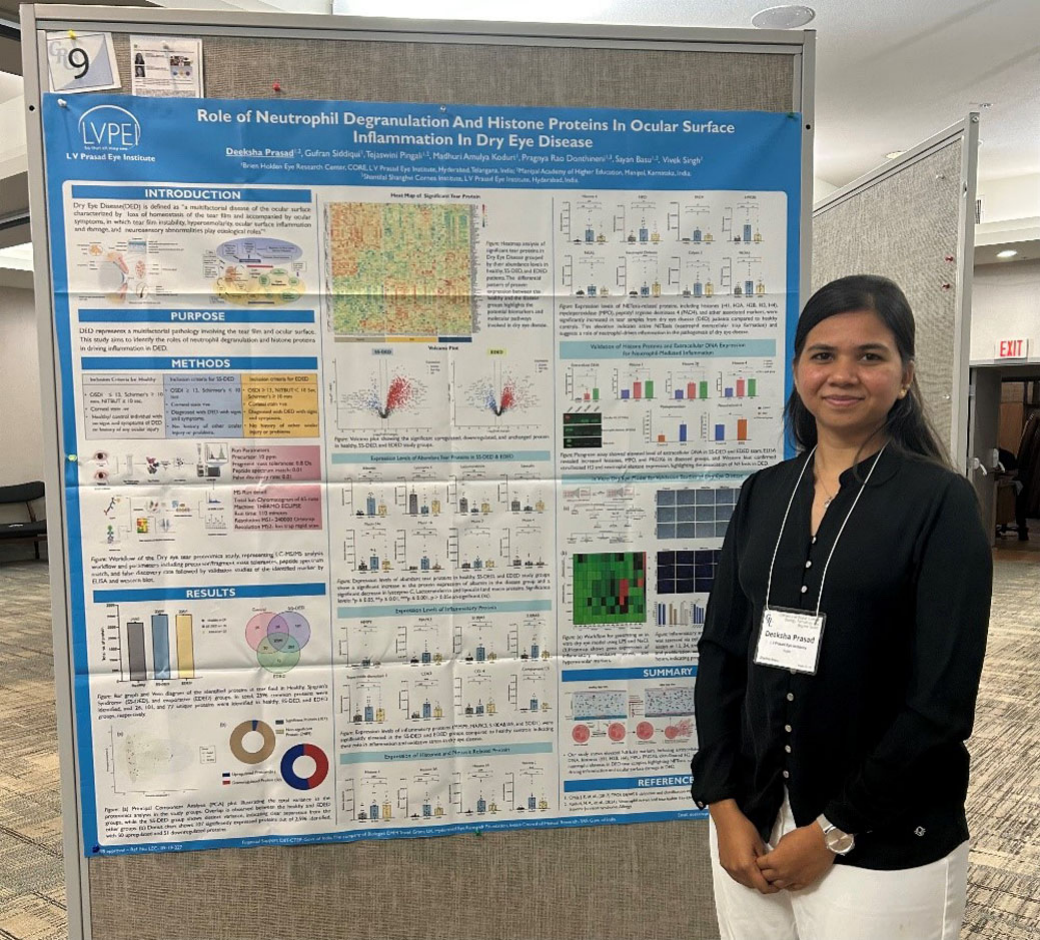
Conference Travel Grant recipient Deeksha Prasad presenting a poster at the ‘2025 Cornea and Ocular Surface Biology, Pathology and Regeneration’ Gordon Research Conference.

Deeksha Prasad (LV Prasad Eye Institute, India) commented that “[the DMM Conference Travel Grant]… allowed me to connect with leading experts in ocular surface biology, engage in insightful discussions and gain valuable feedback on my work. Conversations with researchers working on inflammation, biomaterials and regenerative medicine have given me fresh perspectives and new ideas that will help shape the next phase of my research. This experience has not only deepened my understanding and opened doors for potential collaborations that could significantly impact my future career” (see Fig. 2).

Collaboration and networking at conferences, much missed during the COVID pandemic (Nicholson and Hmeljak, 2020), were also frequently mentioned. The importance of conferences to bring together researchers from around the world was abundantly clear in many reports, and should also be instructive to senior scientists about the value of their spending time at conferences with ECRs.

This was nicely illustrated by Liudmila Saveleva (University of Eastern Finland, Finland), who attended the 8th edition of the Advanced Epilepsy Course Bridging Basic with Clinical Epileptology: “As someone relatively new to epileptology, this course was invaluable. I met experts from all continents. Their openness and advice were incredibly motivating, and I received direct feedback on my current research and future plans … I believe all these new connections will be crucial for future collaborations and shaping my career in epilepsy research”.

Hedda Michelle Guevara-Nieto (Universidad Nacional de Colombia, Colombia) went to the 2025 American Association for Cancer Research (AACR) Annual Meeting, which “offered opportunities for networking with professionals from across fields. These connections proved invaluable, providing insights into emerging trends, mentorship and opportunities for collaboration. Building relationships with both experts and peers facilitated knowledge sharing and support, while connecting with potential collaborators opened doors to exciting projects and career advancements. The meeting provided a solid foundation for the next phase of my career, offering inspiration and resources to help me achieve my goals. Thanks to the DMM travel grant, I was able to access resources and expertise that are difficult to find in my home country, Colombia. This experience will guide my path toward a successful career in cancer research”.

And a final word from Luisina Onofrio, from Universidad Nacional de Córdoba, Argentina, who attended the Federation of Clinical Immunology Societies (FOCIS) European Advanced Course and Conference on Immunology and Immunopathology 2025, in Rouen, France: “One of the highlights of this experience was the opportunity to meet and exchange ideas with young immunologists from 22 different countries. I was amazed by how similar our scientific challenges are, despite coming from very different backgrounds. I had inspiring conversations with colleagues working on autoimmune diseases, cancer immunotherapy and mucosal immunity, which sparked new ideas for future collaborations. Beyond the scientific program, the social events created an ideal setting for networking and friendship … These moments reminded me that science is also about community, empathy and curiosity. This experience has broadened my scientific vision, strengthened my international network, and renewed my motivation to pursue translational immunology research”.

These are just a small sample of the hundreds of reports that DMM has received. Given the clear value of Conference Travel Grants to the ECR community, in 2025, DMM made the decision to focus its charitable giving on these grants, feeling that this is the best mechanism by which DMM can support its ECR community. Support for the ECR community will continue in other ways as well.

## Outstanding Paper Prize

The DMM Outstanding Paper Prize was set up to inspire young researchers embarking on their scientific careers. Each year, the journal awards a prize (£1000) to the first author(s) of the Research and Resources & Methods articles that are judged by the journal's editors to be the most outstanding contribution to the journal that year.

Looking back at recipients, it is gratifying to see many of them progressing in their scientific careers. Our first winner, back in 2018, Dr Wenqing Zhou (Zhou et al., 2018; Justice, 2019), now runs her own lab group, having joined UMass Chan Medical School as a tenure-track assistant professor in July 2025. Our 2019 winner, Alessandro Bailetti (Bailetti et al., 2019; Hackett, 2020), then at Stanford University, is now a biology professor at Evergreen Valley College (San Jose, CA, USA). After receiving her PhD with Dr Griffin's group at the University of Oklahoma, from where she published her winning 2020 DMM paper (Colijn et al., 2020; Hackett, 2021), Sarah Colijn began a postdoctoral position at Washington University in St Louis with Dr Amber Stratman, where we are certain she will fulfil her aim to “carry on the legacy of outstanding female mentorship she has received from Drs Griffin and Stratman as she continues in her own academic career”.

## First Person interviews

Beyond financial support, our popular First Person interviews enable the first authors of Research and Resources & Methods articles published in DMM to talk about their work in and out of the lab, the journeys that led them to where they are now and the issues they feel are priorities for ECRs. Since their launch, DMM has published more than 300 First Person interviews (some of our interviewees are featured on the cover of this issue). The picture that emerges is of a hard-working cohort of researchers dedicated to improving human health globally. These researchers are often inspired by the experiences of family members and have a genuine passion for disease biology research, leaving readers of their interviews with the impression that the future of disease biology research is in safe hands.

## Final remarks

The examples above represent just a few of the ways in which The Company of Biologists and DMM have, and continue to, support ECRs (Bray et al., 2025; https://www.biologists.com/about-us/early-career/). As the Company moves into the decade beyond its 100th birthday in 2025, we remain deeply committed to supporting the research community and fostering the next generation of scientists.
